# Utility of the Apparent Diffusion Coefficient in the Differential Diagnosis of Oral Malignant Lymphoma

**DOI:** 10.7759/cureus.97006

**Published:** 2025-11-16

**Authors:** Hideaki Hirai, Yutaka Nikkuni, Ryota Kobayashi, Tetsuo Kiguchi, Yoshimasa Sumita, Yuko Saito, Atsushi Uenoyama, Akinori Funayama, Kei Tomihara

**Affiliations:** 1 Division of Oral and Maxillofacial Surgery, Faculty of Dentistry ＆ Graduate School of Medical and Dental Sciences, Niigata University, Niigata, JPN; 2 Division of Oral and Maxillofacial Radiology, Faculty of Dentistry ＆ Graduate School of Medical and Dental Sciences, Niigata University, Niigata, JPN; 3 Division of Oral and Maxillofacial Surgery, Faculty of Dentistry & Graduate School of Medical and Dental Sciences, Niigata University, Niigata, JPN; 4 Division of Reconstructive Surgery for Oral and Maxillofacial Region, Faculty of Dentistry ＆ Graduate School of Medical and Dental Sciences, Niigata University, Niigata, JPN

**Keywords:** apparent diffusion coefficient (adc), diffusion-weighted magnetic resonance imaging, malignant lymphoma, oral neoplasm, salivary gland carcinoma, squamous cell carcinoma (scc)

## Abstract

Objective: Malignant lymphoma (ML), a lymphocyte-derived malignancy, is classified into Hodgkin lymphoma and non-Hodgkin lymphoma (NHL). In the oral cavity, NHL is the predominant type. However, diagnosing oral ML is challenging owing to its clinical similarity to other lesions. Diffusion-weighted magnetic resonance imaging and apparent diffusion coefficient (ADC) values may aid in differential diagnosis. Therefore, in the present study, we aimed to evaluate the usefulness of ADC in distinguishing ML from squamous cell carcinoma (SCC) and salivary gland carcinoma (SGC).

Methods: This retrospective study included 11 patients with ML, 35 with SCC, and 19 with SGC treated at our institution between 2012 and 2022. Clinical data and ADC values were analyzed, and magnetic resonance imaging was performed using a 3.0 T scanner.

Results: Clinical assessment alone frequently resulted in misclassification of ML cases, with most cases initially suspected of having benign or other malignant tumors. ML demonstrated markedly lower ADC values (mean 0.67 ± 0.13 × 10⁻^3^ mm^2^/s; range 0.45-0.87) than SCC (1.21 ± 0.14) and SGC (1.30 ± 0.27) (both *P* < 0.0001), with minimal overlap between the groups. An ADC cutoff of 0.87 × 10⁻^3^ mm^2^/s identified ML with 100% sensitivity and 98.2% specificity (area under the curve: 0.99), indicating a strong diagnostic differentiation from carcinoma cases.

Conclusion: ADC assessment may facilitate early suspicion of oral ML and help clinicians in prioritizing it within the differential diagnosis. Integrating ADC values into the diagnostic workflow could facilitate early recognition and more appropriate biopsy strategies, thus supporting timely management.

## Introduction

Malignant lymphoma (ML) refers to lymphocyte-derived malignant tumors, broadly classified into Hodgkin lymphoma and non-Hodgkin lymphoma (NHL). ML can also be categorized based on its site of origin as nodal (arising in lymph nodes) or extranodal (arising in organs or tissues outside the lymphatic system). Although NHL is rarely observed in the oral cavity, it is the most common type of lymphoma in this region, accounting for approximately 2% of all extranodal lymphomas [1,2]. Overall, lymphomas are the third most common malignant tumors in the oral and maxillofacial region, following squamous cell carcinoma (SCC) and salivary gland neoplasms [3]. However, extranodal lymphomas of the oral cavity constitute fewer than 5% of all oral malignant neoplasms [4]. Diagnosis is often challenging owing to their rarity and clinical resemblance to inflammatory and other neoplastic lesions.

As early detection is crucial for improving outcomes, imaging techniques play an essential role in diagnosis. Among these techniques, diffusion-weighted magnetic resonance imaging quantifies the random motion of water molecules within tissues by measuring the apparent diffusion coefficient (ADC). Increased cellularity or swelling, features of many malignant tumors, restrict water diffusion and lead to lower ADC values [5]. Accordingly, malignant tumors generally exhibit lower ADC values than benign or inflammatory lesions [6,7].

In the present study, we aimed to evaluate the diagnostic utility of ADC values in differentiating oral ML from SCC and salivary gland carcinoma (SGC). We hypothesized that ADC values in ML would be significantly lower than those in SCC and SGC. The specific aim of this study was to compare ADC values of ML, SCC, and SGC to determine their potential utility in differential diagnosis.

## Materials and methods

Study design

This retrospective case-control study was conducted at the Department of Oral Surgery, Niigata University Medical & Dental Hospital in Japan, and included patients treated between January 2012 and December 2022. The study population comprised individuals diagnosed with oral ML, SGC, or SCC. Patients with ML or SGC were identified from cases between 2012 and 2022, whereas SCC cases were selected from patients treated between January 2019 and December 2022 to ensure consistency in imaging protocols.

The study included only patients with histopathologically confirmed ML, SCC, or SGC who underwent pretreatment magnetic resonance imaging (MRI) with diffusion-weighted imaging (DWI). Patients without MRI findings were excluded. In addition, cases with motion artifacts that interfered with ADC measurement ​​were also excluded. This study was approved by the Institutional Review Board of Niigata University (approval no. 2023-0092) and adhered to the Helsinki Declaration. The requirement for informed consent was waived owing to the retrospective design and use of anonymized data.

Variables

The primary predictor variable was the ADC value (×10⁻^3^ mm^2^/s), derived from DWI sequences. The primary outcome was the diagnostic ability of ADC values to distinguish ML from SCC and SGC, as measured by classification performance metrics such as sensitivity, specificity, and area under the curve (AUC).

Secondary variables included demographic data, including sex (binary) and age (continuous); anatomic data, such as the primary lesion subsite (categorical); histopathological finding, including lymphoma or carcinoma subtype (categorical); clinical (ML only) data, such as symptom presentation (e.g., swelling, ulceration, pain, tissue elasticity) (categorical); and hematological markers, including lactate dehydrogenase and soluble interleukin-2 receptor (continuous).

Data collection

Demographic, clinical, histopathological, and imaging data were extracted from electronic medical records. MRI was performed using a 3.0 T system (Discovery 750w 3.0T; GE Healthcare, Chicago, IL, USA). ADC maps were generated from DWI sequences with b-values ​​of 0 and 900 s/mm2. Imaging parameters included: repetition time, 7000 ms; echo time, 70 ms; slice thickness, 5 mm; slice interval, 6 mm; and field of view, 26.4 × 23.0 cm. The imaging range extended from the infraorbital rim to the submental region.

Additional sequences included iterative decomposition of water and fat with echo asymmetry and least-squares estimation (IDEAL) T2-weighted, T1-weighted, dynamic contrast-enhanced (7-slice axial), and postcontrast IDEAL T1-weighted images. Axial images were acquired with 5 mm slices and 6 mm intervals; coronal images were acquired with 4 mm slices and 5 mm intervals.

ADC values ​​were calculated by a board-certified oral and maxillofacial radiologist with 18 years of experience. A region of interest covering the entire tumor was delineated on each ADC map slice, and the mean ADC value was calculated for each lesion. To minimize the influence of metal-induced susceptibility artifacts, the diffusion-weighted images used to generate the ADC maps were carefully reviewed. Cases in which artifacts affected the tumor area were excluded from analysis.

Data analysis

The Mann-Whitney U test was used to compare ADC values among the ML, SCC, and SGC groups. Receiver-operating characteristic (ROC) curve analysis was conducted to evaluate diagnostic performance and determine an optimal cutoff value for distinguishing ML. A two-sided P-value < 0.05 was considered statistically significant. Variables with missing data, such as sIL-2R (available in eight of eleven ML cases), were summarized based on available data without imputation. Statistical analyses were performed using JMP Pro for Windows, version 18.1.0 (SAS Institute Inc., Cary, NC, USA).

## Results

Study participants

**Figure 1:**
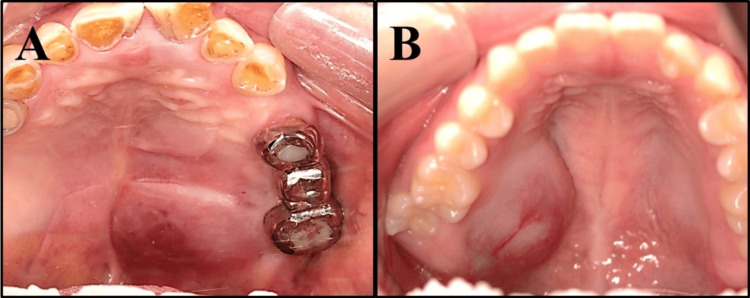
Macroscopic view of the tumor Macroscopic view of a representative tumor highlighting the difficulty in distinguishing between the two lesions based on clinical findings. Diffuse large B cell lymphoma (A) and mucoepidermoid carcinoma (B).

A total of 65 patients were included in the study, including 11 patients with ML, 35 with SCC, and 19 with SGC. Figure 1 presents a case where distinguishing ML based on clinical findings was challenging. Table 1 summarizes the clinical and pathological characteristics of the study groups. All three groups predominantly comprised women. Patients with ML were older (mean age, 80 years) than those with SCC (mean age, 70 years) or SGC (mean age, 62 years). In terms of tumor location, ML most frequently occurred in the maxillary gingiva (36.3%, 4/11 patients), SCC in the tongue (37.1%, 13/35 patients), and SGC in the buccal mucosa and palate (31.6%, 6/19). The predominant histopathological subtype of ML was diffuse large B cell lymphoma (DLBCL) (81.8%, 9/11), whereas mucoepidermoid carcinoma accounted for 52.7% (10/19) of SGC cases.

**Table 1:** Demographic data of all patients DLBCL, diffuse large B cell lymphoma; ML, malignant lymphoma; SCC, squamous cell carcinoma; SGC, salivary gland carcinoma

Demographic data	ML (n=11)	SCC (n=35)	SGC (n=19)
Sex (M/F)			
Male	3 (27.3%)	15 (42.9%)	8 (42.1%)
Female	8 (72.7%)	20 (57.1%)	11 (57.9%)
Age (years)			
Mean	80	70	62
Range	70-93	38-92	14-93
Primary site			
Tongue	0 (0%)	13 (37.1%)	0 (0%)
Lower gingiva	1 (9.1%)	6 (17.1%)	1 (5.2%)
Buccal mucosa	1 (9.1%)	8 (22.9%)	6 (31.6%)
Upper gingiva	4 (36.3%)	6 (17.1%)	3 (15.8%)
Palate	3 (27.3%)	1 (2.9%)	6 (31.6%)
Floor of mouth	2 (18.2%)	1 (2.9%)	0 (0%)
Sublingual gland	0 (0%)	0 (0%)	3 (15.8%)
Histopathological diagnosis			
DLBCL	9 (81.8%)	－	－
Follicular lymphoma	2 (18.2%)	－	－
SCC	－	35 (100%)	－
Mucoepidermoid carcinoma	－	－	10 (52.7%)
Adenoid cystic carcinoma	－	－	4 (21.1%)
Adenocarcinoma, NOS	－	－	2 (10.5%)
Clear cell carcinoma	－	－	2 (10.5%)
Salivary duct carcinoma	－	－	1 (5.2%)

No participants were excluded owing to incomplete data. All participants underwent pretreatment MRI with DWI sequences. No motion artifacts were observed in any of the cases in this study.

Clinical and hematological characteristics of patients with ML

Table 2 summarizes the clinical and hematological findings in patients with ML. The initial clinical diagnosis was ML in two patients (18.2%), benign or salivary gland tumors in six patients (54.5%), and other malignant tumors in three patients (27.3%). All patients presented with swelling; ulceration and pain were observed in three (27.3%) and four (36.3%) cases, respectively. On palpation, lesion consistency was elastic-hard in seven (63.7%) cases and elastic-soft in four cases (36.3%). ADC values ranged from 0.45 to 0.87 ​​× 10^-3^ mm^2^/s (mean, 0.67; standard deviation [SD], 0.13). Positron emission tomography (PET) was performed in four patients, yielding maximum standardized uptake values ranging from 19.6 to 33 (mean, 25.8; SD, 5.4). Elevated lactate dehydrogenase levels were observed in 1 of 11 participants (9.1%), whereas elevated soluble interleukin-2 receptor (sIL-2R) levels were noted in 7 of 8 participants (87.5%). sIL-2R values were available for eight patients; three with missing data were excluded from this descriptive summary.

**Table 2:** Demographic and clinical features of ML patients M, male; F, female; FOM, floor of mouth; ML, malignant lymphoma; FL, follicular lymphoma; DLBCL, diffuse large B cell lymphoma; MRI, magnetic resonance imaging; ADC, apparent diffusion coefficient; PET, positron emission tomography; SUV, standardized uptake value; LDH, lactate dehydrogenase; sIL-2R, soluble interleukin-2 receptor Reference range: LDH, 124–222 U/L; sIL-2R, 204–587 U/mL

Case	Gender	Age (yrs)	Primary site	Initial clinical diagnosis	Histopathological diagnosis	Swelling	Ulceration	Pain	Elasticity	MRI ADC(x10^-3^mm^2^/s)	PET SUV_max_	LDH (U/L)	sIL-2R (U/mL)
1	M	71	Palate	Benign tumor	FL	+	－	－	Hard	0.87	－	170	660
2	F	78	FOM	Schwannoma	DLBCL	+	－	－	Hard	0.79	－	193	－
3	F	71	Palate	Salivary gland tumor	DLBCL	+	－	－	Soft	0.6	－	169	243
4	M	88	Upper gingiva	Benign tumor	DLBCL	+	－	－	Soft	0.75	－	150	6917
5	F	83	Upper gingiva	Benign tumor	DLBCL	+	－	－	Soft	0.8	－	209	4260
6	F	70	Upper gingiva	ML	DLBCL	+	+	+	Hard	0.65	28.9	222	765
7	F	83	Lower gingiva	Carcinoma	DLBCL	+	+	+	Hard	0.58	21.5	171	859
8	F	80	Upper gingiva	Carcinoma	DLBCL	+	－	－	Hard	0.54	19.6	192	－
9	M	88	Buccal mucosa	Salivary gland tumor	DLBCL	+	－	+	Hard	0.45	－	351	1430
10	F	93	FOM	Carcinoma	DLBCL	+	+	+	Hard	0.77	33	187	10371
11	F	75	Palate	ML	FL	+	－	－	Soft	0.56	－	179	－

ADC values

**Figure 2:**
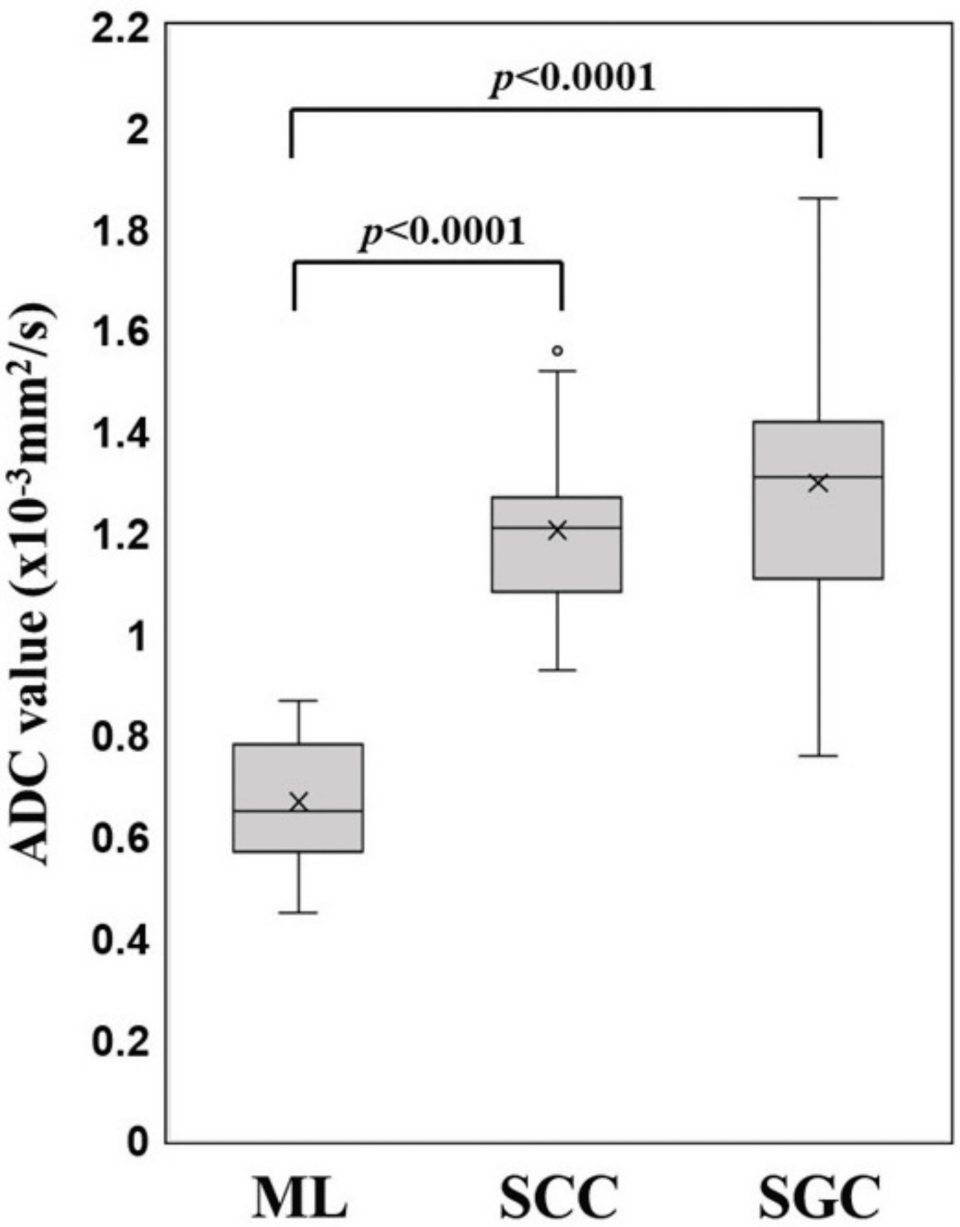
Comparison of ADC values between ML, SCC, and SGC ADC values in ML were significantly lower than those in SCC and SGC (P < 0.0001 for both). ADC, apparent diffusion coefficient; ML, malignant lymphoma; SCC, squamous cell carcinoma; SGC, salivary gland carcinoma

Figure 2 depicts the ADC distribution across tumor types. The mean ADC value for ML was significantly lower than those for SCC and SGC: ​​ML: 0.67 ± 0.13 × 10^-3^ mm^2^/s; SCC: 1.21 ± 0.14 × 10^-3^ mm^2^/s; SGC: 1.3 ± 0.27 × 10^-3^ mm^2^/s. Moreover, comparisons between ML and both carcinoma groups were statistically significant (P < 0.0001 for both). Among ML subtypes, the mean ADC value for DLBCL (n = 9) was 0.66 × 10^-3^ mm^2^/s, whereas that for follicular lymphoma (FL) (n = 2) was 0.72 × 10^-3^ mm^2^/s. However, this difference was not statistically significant (P > 0.10). Finally, ROC analysis identified an optimal ADC cutoff of 0.87 × 10^-3^ mm^2^/s for distinguishing ML from SCC and SGC, yielding 100% sensitivity, 98.2% specificity, and an AUC of 0.99.

## Discussion

The present study evaluated the diagnostic utility of ADC values in differentiating oral ML from SCC and SGC. We hypothesized that ADC values in ML would be significantly lower than those in SCC and SGC. Our results support this hypothesis, as the mean ADC value of oral ML was significantly lower than that of SCC and SGC, and a cutoff value of 0.87 × 10⁻³ mm²/s demonstrated excellent diagnostic performance (sensitivity 100%, specificity 98.2%, AUC 0.99).

Oral ML exhibits few distinctive clinical features, making clinical differentiation from other tumor-like lesions extremely difficult [8,9]. In our study, swelling was observed in all cases, whereas ulceration and pain were present in some, and palpation revealed variable lesion consistency. Although sIL-2R levels exceeded the reference range in most cases, this marker is not specific for ML and may also be elevated in infections or renal failure [4,8]. At the time of initial clinical diagnosis, only two patients (18.2%) were correctly diagnosed with ML, whereas more than half were initially diagnosed with benign or salivary gland tumors. These findings highlight the diagnostic challenge of oral ML.

DWI, a rapid sequence derived from echo-planar and spin-echo imaging [10], enables quantification of water molecule motion in tissue through ADC measurements. It is valuable for evaluating oral and maxillofacial lesions. In general, ADC values ​​are lower in highly cellular tissues, making DWI useful for tumor characterization and for diagnosing cerebral ischemia [11,12]. Malignant tumors typically exhibit lower ADC values than benign or inflammatory lesions [6,7]. Moreover, ML usually demonstrates lower ADC values than metastatic lymph nodes or benign lymphadenopathy [13-15]. Among malignant tumors, ML has consistently been reported to exhibit particularly low ADC values [16,17]. Our study confirmed that ADC values ​​in oral ML were significantly lower than those in SCC, which is the most common oral malignancy, and in SGC, which presents a challenging differential diagnosis. Wang et al. reported ADC values ​​in head and neck lesions as follows: ML, 0.66 ± 0.17 × 10^-3^ mm^2^/s; carcinoma, 1.13 ± 0.43 × 10^-3^ mm^2^/s; benign solid mass, 1.56 ± 0.51 × 10^-3^ mm^2^/s; benign cystic lesion, 2.05 ± 0.62 × 10^-3^ mm^2^/s [16], with significant differences observed between ML and all other lesion types (P < 0.001). Similarly, Shiraishi et al. reported significant differences between ML and SCC in the head and neck, with ADC values of 0.762 ± 0.126 × 10^-3^ mm^2^/s and 1.24 ± 0.22 × 10^-3^ mm^2^/s, respectively (P < 0.0001) [17]. However, some poorly differentiated carcinomas may exhibit ADC values ​​similar to those of ML, owing to high cellular density, large and angular nuclei, abundance of macromolecular proteins, and limited extracellular space [16]. For example, Hodgkin's lymphoma typically exhibits higher ADC values than NHL [13,15,18]. Conversely, Wu et al. reported no significant difference in ADC values ​​between DLBCL and FL in the context of NHL [19]. Similarly, our study found no statistically significant differences between DLBCL and FL.

An advantage of ADC-based imaging is that, unlike CT or PET, MRI does not involve radiation exposure, making it especially suitable for children and younger patients. In ML, ADC values have been reported to predict treatment response [20,21]. For example, Chien et al. reported that pretreatment ADC values and response to first-line chemotherapy were significant predictors of clinical outcomes in primary central nervous system lymphoma [21]. Similarly, our results suggest that ADC values may contribute to early treatment planning in oral ML. In our study, definitive diagnoses were established by biopsy, and patients were referred to a hematologist for further management. In such cases, tissue sampling for flow cytometry may also be warranted. Our results suggest that if the ADC value ​​is below 0.87 × 10^-3^ mm^2^/s, incorporating flow cytometry at the time of initial biopsy may help facilitate earlier treatment. However, a limitation of this approach is that patients with pacemakers, cochlear implants, or internal metallic devices cannot undergo MRI and, therefore, cannot undergo ADC measurements.

This study has several limitations. First, the sample size was small, owing to the rarity of oral ML. Second, we did not analyze ADC values by histological grade in SCC, as only one case of poorly differentiated carcinoma was included. Similarly, we did not evaluate ADC values ​​by histological subtype in SGC. Given the diversity of SGC subtypes, differences in ADC values may exist depending on histological grade, malignancy potential (e.g., mucoepidermoid carcinoma), and variants such as adenoid cystic carcinoma. Third, variations in MRI parameters across institutions, including field strength and imaging protocols, may limit the generalizability of our ADC reference values. Fourth, ADC measurements were performed by a single observer, which may have introduced bias. However, the measurement range on the image was set by a single observer, and the standard was consistent. Finally, ADC values may be influenced by patient age and tumor location, but due to the small number of cases, these effects were not examined. Future studies with larger sample sizes are warranted to validate these findings and to clarify the potential effects of age and tumor site on ADC values. Despite these limitations, the present results provide a useful foundation for further investigation.

## Conclusions

This study demonstrated that ADC values obtained from diffusion-weighted MRI provide a clinically actionable imaging marker for raising early suspicion of oral ML, a tumor that often lacks distinctive clinical features and is frequently misdiagnosed at initial presentation. Moreover, incorporating ADC assessment into routine MRI interpretation may help clinicians prioritize ML within the differential diagnosis and guide biopsy strategies, including the timely use of flow cytometry when ML is suspected. This approach may also support earlier recognition and streamline the diagnostic pathway. However, further multicenter validation is warranted to establish standardized ADC thresholds and promote broader clinical integration.
